# Whole transcriptome and proteome analyses identify potential targets and mechanisms underlying tumor treating fields against glioblastoma

**DOI:** 10.1038/s41419-022-05127-7

**Published:** 2022-08-18

**Authors:** Shengchao Xu, Chengke Luo, Dikang Chen, Lu Tang, Ling Chen, Zhixiong Liu

**Affiliations:** 1 Department of Neurosurgery, Xiangya Hospital, Central South University, Changsha, 410008 Hunan China; 2 National Clinical Research Center for Geriatric Disorders, Xiangya Hospital, Central South University, Changsha, China; 3 Hunan An Tai Kang Cheng Biotechnology Co., Ltd, Changsha, China; 4 Department of Anesthesiology, Xiangya Hospital, Central South University, Changsha, 410008 Hunan China; 5 Department of Neurosurgery, Chinese People’s Liberation Army of China (PLA) General Hospital, Medical School of Chinese PLA, Institute of Neurosurgery of Chinese PLA, 100853 Beijing, China

**Keywords:** CNS cancer, Cancer therapy

## Abstract

Glioblastoma (GBM) is one of the most malignant types of brain cancer. Tumor treating fields (TTFields) is the up-to-date treatment for GBM. However, its molecular mechanism requires additional investigation. Herein, a novel TTFields system was developed (CL-301A) and its efficiency in suppressing GBM cell proliferation and inducing cell apoptosis was demonstrated. Through the whole proteomic and transcriptomic analyses, a multitude of differentially expressed proteins (1243), mRNAs (4191), miRtNAs (47), lncRNAs (4286), and circRNAs (13,903) were identified. Bioinformatic analysis indicated that TTFields mainly affected nuclear proteins and interrupt cell mitosis-related events. Moreover, the inhibition of autophagy could significantly enhance the anti-GBM activity of TTFields. And CDK2-AS1 might be a target of TTFields to mediate cell cycle arrest via regulating CDK2 mRNA stability. This study provided valuable resources for understanding the mechanism of TTFields, which might further assist the investigation of TTFields in GBM treatment.

## Introduction

Gliomas are the most common primary brain cancer that account for more than 80% of brain cancers [1]. Based on the World Health Organization (WHO) classification, gliomas are histologically classified into four grades and the higher grade indicates worse prognosis [2]. Glioblastoma (GBM) belongs to grade IV and is the most malignant subtype of gliomas. The median overall survival (OS) of GBM patients is 14 months and the five-year OS rate is less than 5% [3]. Conventional therapies for GBM include surgery, chemotherapy, and radiotherapy. However, the prognosis of GBM patients remains poor.

Although numerous cancer therapies have been developed in recent years, little progress has been made for the treatment of GBM. Temozolomide (TMZ) is the most commonly used chemotherapy for GBM patients, and its efficacy is established in a clinical trial that the combination of TMZ and radiotherapy significantly prolongs the median OS of GBM patients compared with radiotherapy alone [4]. In additional to TMZ, bevacizumab and tumor-treating fields (TTFields) are the remaining two therapeutics approved for GBM treatment. TTFields is an electric field therapy that can significantly inhibit the proliferation of various tumor cells [5]. In patients with recurrent GBM and received TTFields intervention, the median OS was 62.2 weeks, which was markedly higher than that of historical control patients [6]. The promising efficacy of TTFields conferred the large-scale clinical trial, which demonstrated that the combination of TMZ and TTFields significantly prolonged the progression-free survival and OS of GBM patients compared with TMZ alone [7]. Currently, TTFields has been added to guideline for treatment for GBM patients by the National Comprehensive Cancer Network (NCCN), Chinese Glioma Cooperative Group (CGCG) and Asian Society of Neuro-Oncology (ASNO) [8, 9].

Here, our research team designed and manufactured an TTFields system (CL-301A) that could apply alternative electric fields for GBM treatment. Our previous results have shown the efficacy of CL-301A in primary GBM cells and glioma rat model [10, 11]. However, the molecular mechanisms of TTFields in GBM remain largely unknown. In this study, we conducted whole-transcriptional and whole-proteomic sequencing in GBM cells with TTFileds intervention, aiming to identify potential targets and molecular mechanisms underlying TTFields against GBM.

## Results

### TTFields intervention suppressed GBM cell proliferation and induced cell apoptosis

**Fig. 1:**
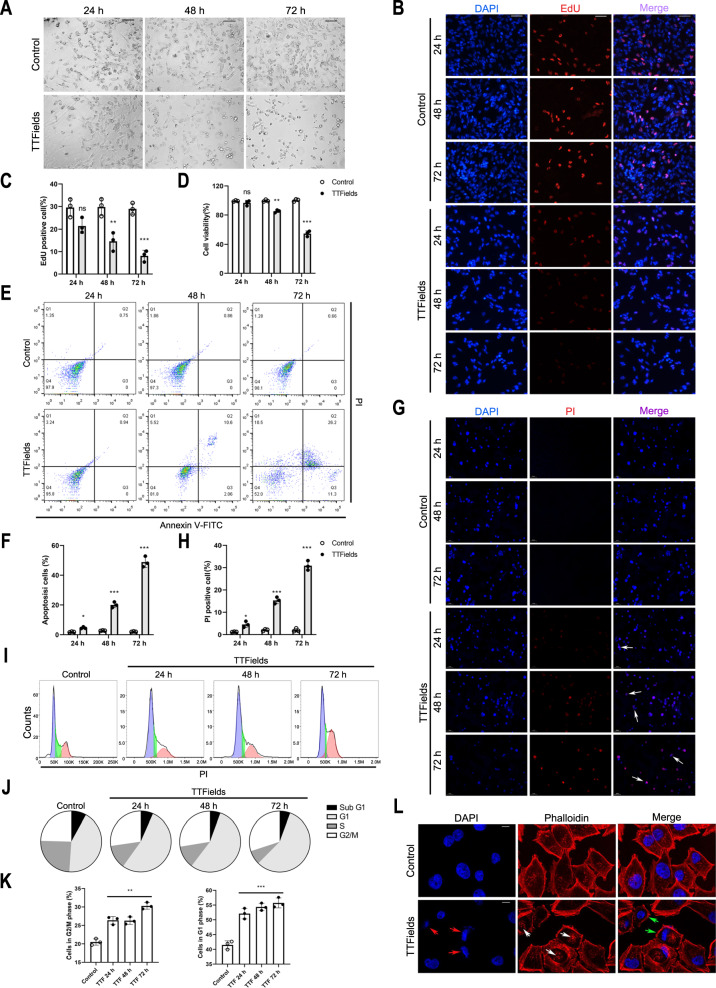
TTFields intervention suppressed GBM cell proliferation and induced cell apoptosis. **A**–**K** Cell morphology (200× magnification) (**A**), EdU assay (scale bar = 50 μm) (**B**, **C**), cell viability (**D**), cell apoptosis assay (**E**, **F**), PI staining (scale bar = 50 μm) (**G**, **H**), and cell cycle analysis (**I**–**K**) of DBTRG cells with TTFields or not for 24 h, 48 h, and 72 h, respectively. **L** Phalloidin staining of DBTRG cells with 48 h TTFields intervention or not. The nuclear aberration was indicated with red arrows, whereas white arrows indicated the accumulations of actin, and green arrows indicated abnormal cell shape (scale bar = 10 μm). *p < 0.05, **p < 0.01, ***p < 0.001.

We firstly explored the effect of TTFields on DBTRG cells and the flow chart of TTFields intervention was shown in Supplementary Fig. S1. Under light microscopy, we found that DBTRG cells became round and presented plasmolysis after TTFields intervention, which aggravated in a time-dependent manner (Fig. 1A). Besides, the proportion of EdU positive cells was significantly reduced after 48-hour and 72-hour TTFields intervention (Fig. 1B, C). Moreover, cell viability was significantly suppressed after 48-hour or 72-hour TTFields intervention (Fig. 1D). Flow cytometry revealed that the percentage of apoptotic cells was significantly promoted by TTFields in a time-dependent manner (Fig. 1E, F). Similarly, TTFields significantly increased the percentage of PI-positive cells in a time-dependent manner (Fig. 1G, H). As for cell cycle, TTFields significantly elevated the percentage of cells arrested at G2/M and G1 phases (Fig. 1I–K). With phalloidine staining, nuclear condensation and cytoskeletal disturbance were detected in DBTRG cells treated with TTFields (Fig. 1L). Therefore, these results preliminarily demonstrated that TTFields could significantly suppress GBM cell proliferation, induce cell cycle arrest and promote cell apoptosis.

### Identification of DEPs, DEmRNAs, DEcircRNAs, DElncRNAs, and DEmiRNAs

**Fig. 2:**
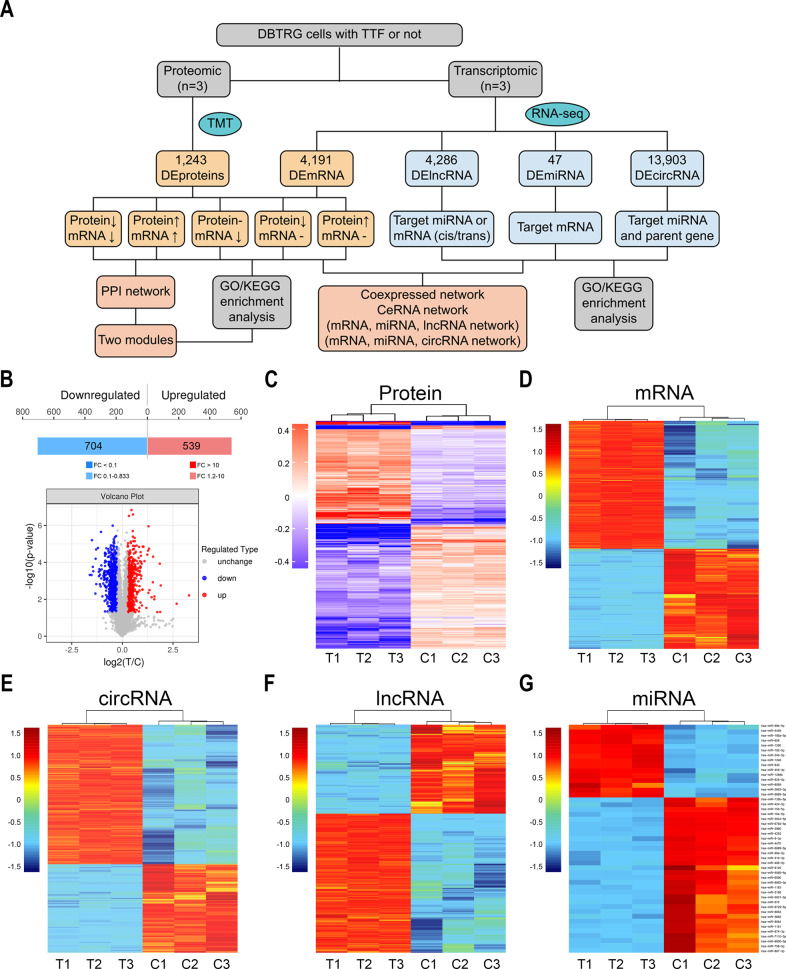
Identification of DEPs, DEmRNAs, DEcircRNAs, DElncRNAs, and DEmiRNAs. **A** Flow chart of transcriptomic and proteomic analysis of DBTRG cells with TTFields or not. **B** The number of DEPs and corresponding volcano plot. Heatmap of DEPs (**C**), DEmRNAs (**D**), DEcircRNAs (**E**), DElncRNAs (**F**), and DEmiRNAs (**G**).

To investigate the underlying mechanisms and potential targets of TTFields, we conducted whole-transcriptional and whole-proteomic sequencing to explore potential targets and molecular mechanisms of TTFields (Fig. 2A). A total of 1243 DEPs including 704 downregulated and 539 upregulated proteins were identified between TTFields and control groups (Fig. 2B, C, Supplementary Table S1). Besides, 4191 DEmRNAs (2352 upregulated and 1839 downregulated, Fig. 2D, Supplementary Table S2), 13,903 DEcircRNAs (8,508 upregulated and 5395 downregulated, Fig. 2E, Supplementary Table S3), 4,286 DElncRNAs (2,613 upregulated and 1673 downregulated, Fig. 2F, Supplementary Table S4), and 47 DEmiRNAs (15 upregulated and 32 downregulated, Fig. 2G, Supplementary Table S5) were identified between TTFields and control groups. As shown in the heatmap, the intervention group was significantly separated from the control group.

### Functional enrichment of DEPs

**Fig. 3:**
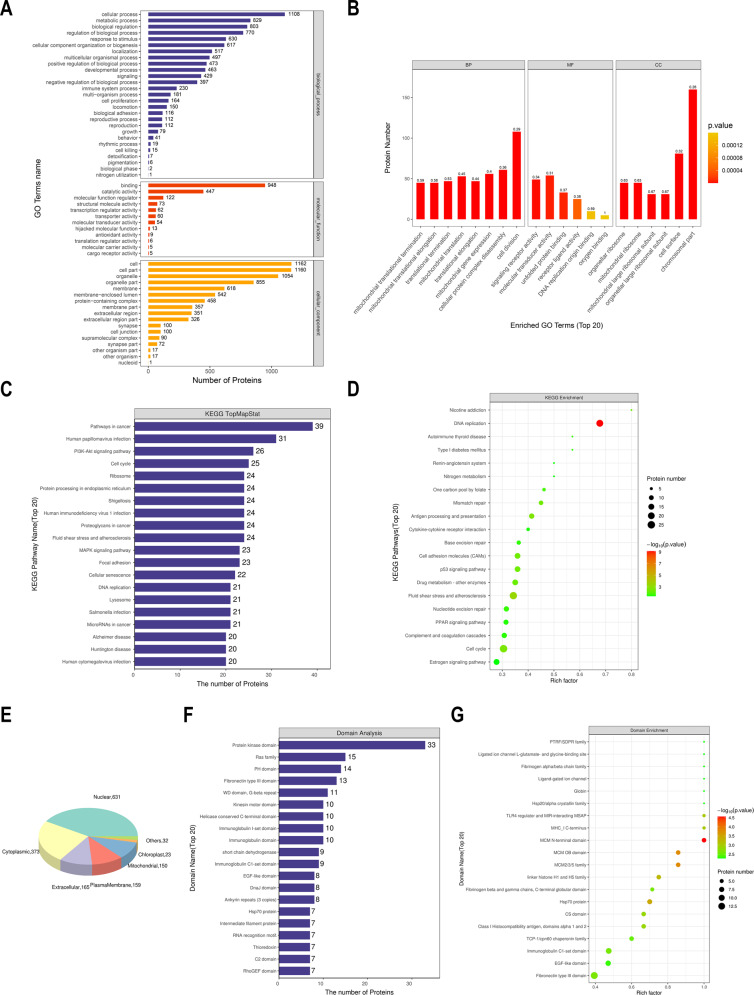
Functional enrichment of DEPs. GO (**A**, **B**) and KEGG (**C**, **D**) enrichment analyses of DEPs. **E** The distribution of subcellular location of DEPs. **F**, **G** Enrichment analysis of domains of DEPs.

Further, we explored the biological activities of DEPs between TTFields and control groups. In biological process, a large number of DEPs were involved cellular process, metabolic process, biological regulation, cell proliferation, locomotion, etc.; as for molecular function, they were enriched in cell binding, catalytic activity, transcription regulator activity, transporter activity, etc.; in cellular component, they were associated with organelle, membrane-enclosed lumen, synapse, cell junction, etc. (Fig. 3A). Besides, they were significantly associated with mitochondrial activities, cell division, translational regulation, DNA replication, and ribosome structures (Fig. 3B). As for pathway analysis, DEPs were associated with cancer, PI3K-Akt pathway, cell cycle, MAPK signaling pathway, ribosome, DNA replication, lysosome, mismatch repair, p53 signaling pathway, etc. (Fig. 3C, D). Then we predicted the cellular location of DEPs, whose majority was located in nucleus (Fig. 3E). Meanwhile, the domains of DEPs mainly enriched with kinase domain, Ras family, EGF-like domain, etc. (Fig. 3F, G). Together, these DEPs were associated with cell proliferation, diverse cellular processes, and multiple organelle activities and structures.

### Functional enrichment of parent genes of DEcircRNAs and target genes of DEmiRNAs and DElncRNAs

To comprehensively investigated the functions of differentially expressed non-coding RNAs, we conducted enrichment analyses of parent genes of DEcircRNAs and target genes of DEmiRNAs and DElncRNAs. The parent genes of DEcircRNAs were enriched in p53 signaling pathway, glioma, DNA replication, cell cycle, lysosome, etc. (Supplementary Fig. S2A). The target genes od DEmiRNAs were associated with Ras siglaing pathway, PI3K-Akt signaling pathway, p53 signaling pathway, cellular senescence, etc. (Supplementary Fig. S2B). Moreover, DElncRNAs cis-regulated genes were involved in glioma, ErbB signaling pathway, F-actin capping protein complex, etc. (Supplementary Fig. S2C). Those DElncRNAs trans-regulated genes were correlated with glioma, p53 signaling pathway, lysosome, HIF-1 signaling pathway, mitotic cell cycle, etc. (Supplementary Fig. S2D). Together, these genes were associated with glioma, p53 signaling pathway, and cell cycle.

### Functional enrichment of co-expressed DEPs-DEmRNAs

Considering the intimate correlation between mRNA and protein, we conducted enrichment analyses to characterize those DEPs and DEmRNAs that shared similar expression patterns. Firstly, these DEmRNAs were associated with biological regulation, organelle, catalytic activity, etc. (Supplementary Fig. S3A). Besides, they were involved in glioma, cell cycle, p53 signaling pathway, mTOR signaling pathway, etc. (Supplementary Fig. S3B). Among DEPs and DEmRNAs, a total of 148 proteins upregulated in both mRNAs and proteins and 200 proteins downregulated in both mRNAs and proteins were identified (Supplementary Fig. S3C). Enrichment analysis revealed that these proteins were associated with mitotic nuclear division, protein folding, spindle organization, kinetochore, DNS helicase activity, etc. (Supplementary Fig. S3D). Meanwhile, they were significantly associated with cell cycle, DNA replication, and p53 signaling pathway (Supplementary Fig. S3E). Hence, these co-expressed DEPs-DEmRNAs were associated with cell cycle, DNA replication, and organelles that were crucial for cell mitosis.

### Functional enrichment of DEPs-DEmRNAs with different expression patterns

**Fig. 4:**
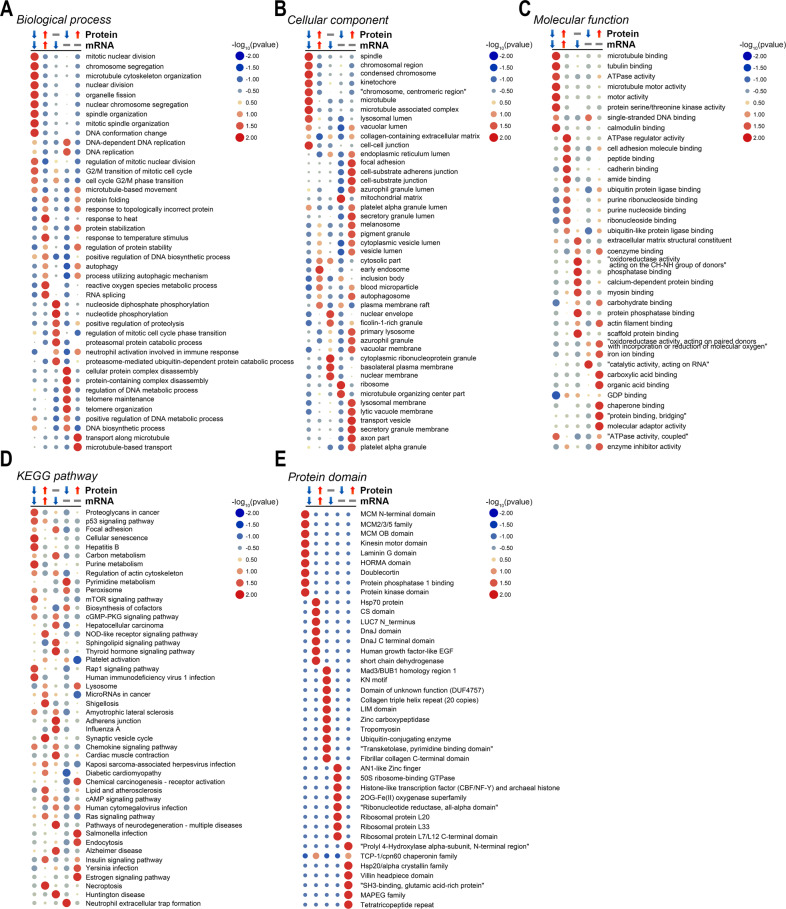
Functional enrichment of DEPs-DEmRNAs with different expression patterns. GO enrichment analysis including biological process (**A**), cellular component (**B**), and molecular function (**C**) and KEGG enrichment analysis (**D**) of DEPs-DEmRNAs with different expression patterns. **E** Protein domain enrichment of DEPs-DEmRNAs with different expression patterns.

Given the fact that post-transcriptional and post-translational modifications would notably affect the level of mRNAs and proteins, we also investigated proteins that exhibited different expression patterns among identified DEPs and DEmRNAs. Apart from 200 downregulated and 148 upregulated proteins as previously mentioned, there were 150 proteins that were d, 174 were unchanged in mRNA but decreased in protein, and 170 were unchanged in mRNA but upregulated in protein (Supplementary Fig. S4). Enrichment analyses revealed that commonly downregulated proteins were associated with mitotic nuclear division, microtubule binding, organelle fission, spindle organization, and p53 signaling pathway, which were critical for cell mitosis (Fig. 4A–D), and they were enriched with protein kinase domain (Fig. 4E). Those commonly upregulated proteins were associated with reactive oxygen species metabolic process, RNA splicing, etc. (Fig. 4A–E). Meanwhile, those decreased in mRNA but unchanged in protein were enriched in cell cycle G2/M phase transition, nuclear envelope, phosphatase binding, etc. (Fig. 4A–E). Moreover, those with unchanged mRNA but decreased protein levels were associated with DNA replication, telomere maintenance, microtubule organization, and neutrophil extracellular trap formation (Fig. 4A–E). Those with unchanged mRNA but increased protein levels were enriched in autophagy, protein folding, autophagosome, and lysosome (Fig. 4A–E). Based on these results, we found that TTFields intervention markedly reduced the level of cell cycle-associated proteins or pathways, and those increased proteins might correlate with cell autophagy.

### Identification of key modules of downregulated proteins and construction of ceRNA network

Since downregulated proteins might be potential targets of TTFields, we constructed the PPI network of the 200 commonly downregulated proteins, in which we identified two key modules (module 1 and module 2) within the PPI network (Supplementary Fig. S5A, B). Enrichment analysis revealed that proteins in module 1 were enriched in cell mitosis and related organelles, whereas those in module 2 were enriched in multiple catabolic and catabolic processes (Supplementary Fig. S5C). Considering that cell cycle was significantly interfered by TTFields, we selected cell cycle-related genes among DEPs-DEmRNAs and constructed the lncRNA-miRNA-mRNA and circRNA-miRNA-mRNA networks (Supplementary Fig. S5D, E).

### Inhibition of autophagy enhanced anti-tumor activity of TTFields against GBM cells

**Fig. 5:**
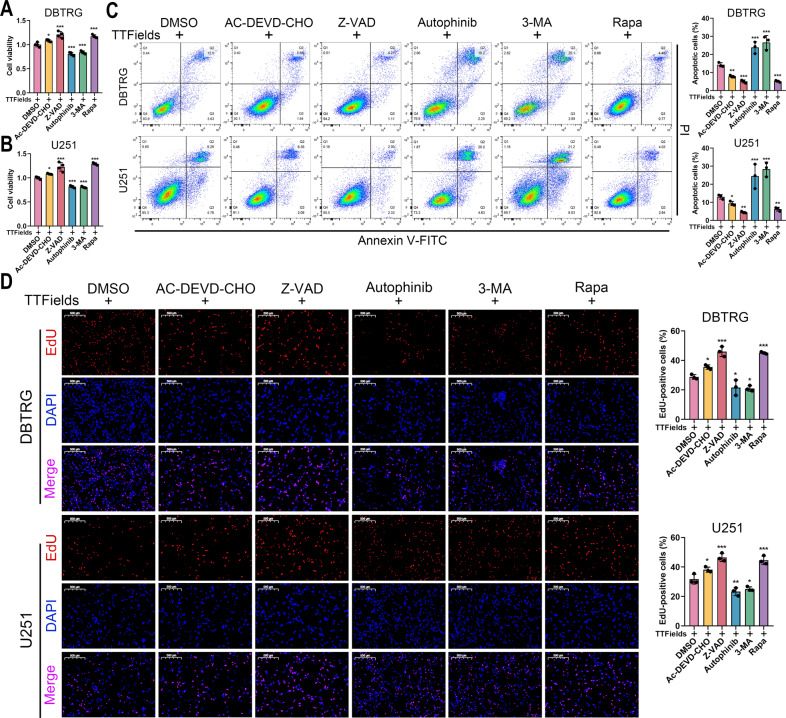
Inhibition of autophagy enhanced anti-tumor activity of TTFields against GBM cells. **A–D** Cell viability (**A**, **B**), cell apoptosis, and Edu assay (scale bar = 500 μm) (**D**) of DBTRG and U251 cells that were pretreated with DMSO, 5 mM AC-DEVD-CHO, 20 mM Z-VAD, 1 μM Autophinib, 5 mM 3-MA, or 100 nM Rapa for 12 h and subsequent TTFields intervention for 48 h. *p < 0.05, **p < 0.01, ***p < 0.001.

**Fig. 6:**
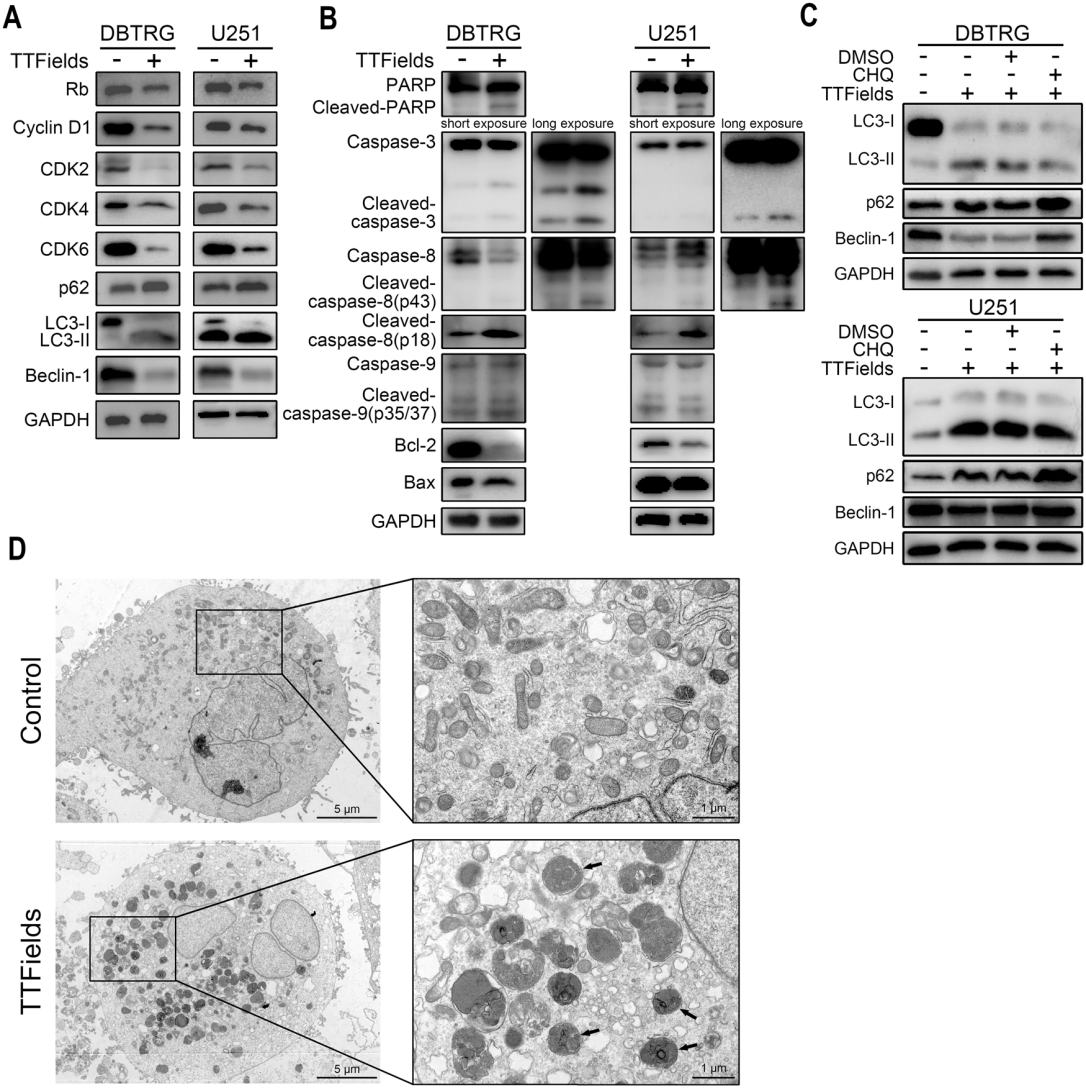
TTFields induced cell apoptosis and suppressed autophagic flux in GBM cells. The protein level of cell cycle-associated proteins and autophagy markers (**A**) and apoptotic markers (**B**) in DBTRG and U251 cells after 48 h TTFields intervention. **C** DBTRG and U251 cells pretreated with 20 μM CHQ for 12 h and then treated with 48 h TTFields, and the expression of autophagy markers were estimated by immunoblot. **D** Transmission electronic microscopy of DBTRG cells treated with TTFields or not (scale bar = 5 μm or 1 μm).

In order to explore the molecular mechanism of TTFields, DBTRG and U251 were pre-treated with several inhibitors or activator following TTFields intervention. As shown in cell morphology, the cytoplasm of DBTRG and U251 cells markedly shrunk in DMSO group after TTFields intervention, which further aggravated in Autophinib and 3-MA groups (Supplementary Fig. S6). In contrast, in Z-VAD and Rapa groups, the majority of cells maintained normal cell morphology as the wildtype group (Supplementary Fig. S6). Further, we found that cell viability was significantly promoted by caspase inhibitors (AC-DEVD-CHO and Z-VAD) and Rapa but decreased by autophagy inhibitors (Autophinib and 3-MA) (Fig. 5A, B). Besides, the percentage of apoptotic cells was significantly decreased by AC-DEVD-CHO, Z-VAD and Rapa but increased by Autophinib and 3-MA (Fig. 5C). Moreover, the percentage of EdU-positive cells was significantly higher in AC-DEVD-CHO, Z-VAD and Rapa groups compared with DMSO group, whereas that was significantly lower in Autophinib and 3-MA groups (Fig. 5D). Western blot revealed that the expression of Rb, Cyclin D1, CDK2, CDK4, and CDK6 was decreased in TTFields group (Fig. 6A, Supplementary Fig. S8). Meanwhile, the expression of p62 and LC3-II/LC3-I was promoted in TTFields group whereas that of Beclin-1 was decreased (Fig. 6A, Supplementary Fig. S8). Moreover, TTFields intervention significantly activated the cleavage of PARP, caspase-3, and caspase-8 and decreased the expression of Bcl-2, however, the cleavage of caspase-9 and the expression of Bax was not markedly affected (Fig. 6B, Supplementary Fig. S9). Since the elevated p62 indicated suppressed autophagic flux and the LC3-II amount could not accurately estimate the autophagic activity [12], we adopted lysosome inhibitor, CHQ, to evaluate the effect of TTFields on autophagy. Results showed that the expression of LC3-II/LC3-I was promoted by TTFields but did not increase by CHQ, whereas the expression of p62 and Beclin-1 was promoted by CHQ (Fig. 6C, Supplementary Fig. S10). Therefore, we proposed that autophagic flux might be interrupted by TTFields. Meanwhile, transmission electron microscope revealed that TTFields notably activated lysosomes but autophagosomes were not detected (Fig. 6D). Consequently, these results indicated that TTFields could decrease autophagic flux and induce cell apoptosis in GBM cells.

### Identification of lncRNA CDK2-AS1 as a potential target of TTFields

**Fig. 7:**
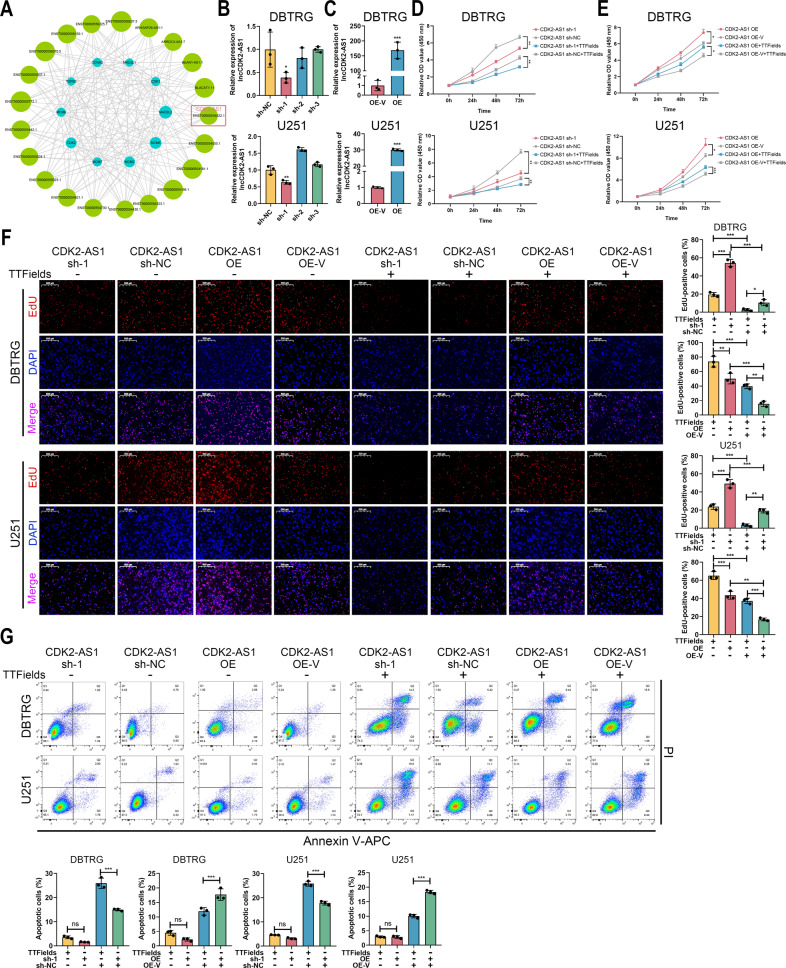
Identification of lncRNA CDK2-AS1 as a potential target of TTFields. **A** Co-expression network of lncRNA and mRNA. **B** CDK2-AS1 expression in DBTRG and U251 cells transfected with sh-1, sh-2, sh-3 or sh-NC. **C** CDK2-AS1 expression in DBTRG and U251 cells transfected with overexpressed plasmid or control vector. **D**–**G** DBTRG and U251 cells transfected with CDK2-AS1 sh-1 or shNC and OE or OE vector were treated with 48 h TTFields or not. CCK-8 assay (**D**, **E**), EdU assay (**F**), and cell apoptosis (**G**) were conducted and estimated. *p < 0.05, **p < 0.01, ***p < 0.001.

Further, we constructed a lncRNA-mRNA co-expression network to identify DElncRNAs that might correlate with cell cycle-associated mRNAs, in which lncRNA antisense to CDK2 (CDK2-AS1, ENST00000554022.1) was selected for further analysis (Fig. 7A). Three shRNAs were designed to inhibit the expression of CDK2-AS1 in DBTRG and U251 cells, in which the sh-1 exhibited the highest efficiency in two cells (Fig. 7B). Besides, the overexpression lentivirus was transduced into DBTRG and U251 cells, which significantly promoted CDK2-AS1 expression (Fig. 7C). The knockdown of CDK2-AS1 significantly reduced GBM cell proliferation, which was further decreased after TTFields intervention (Fig. 7D). In contrast, the overexpression of CDK2-AS1 significantly promoted cell proliferation of DBTRG and U251 cells, and it could partially rescue the therapeutic effect of TTFields (Fig. 7E). Moreover, the knockdown of CDK2-AS1 significantly decreased the percentage of EdU-positive cells, which was further reduced after TTFields intervention (Fig. 7F). Meanwhile, the overexpression of CDK2-AS1 significantly promoted the percentage of EdU-positive cells, which was suppressed by TTFields (Fig. 7F). Additionally, the knockdown or overexpression of CDK2-AS1 did not significantly change the level of cell apoptosis, however, with TTFields intervention, the knockdown of CDK2-AS1 significantly promoted cell apoptosis, whereas its overexpression exerted opposite effects (Fig. 7G). Hence, CDK2-AS1 was identified as a potential target of TTFields to inhibit GBM cell proliferation.

### Knockdown of CDK2-AS1 induced cell cycle arrest via decreasing CDK2 mRNA stability

**Fig. 8:**
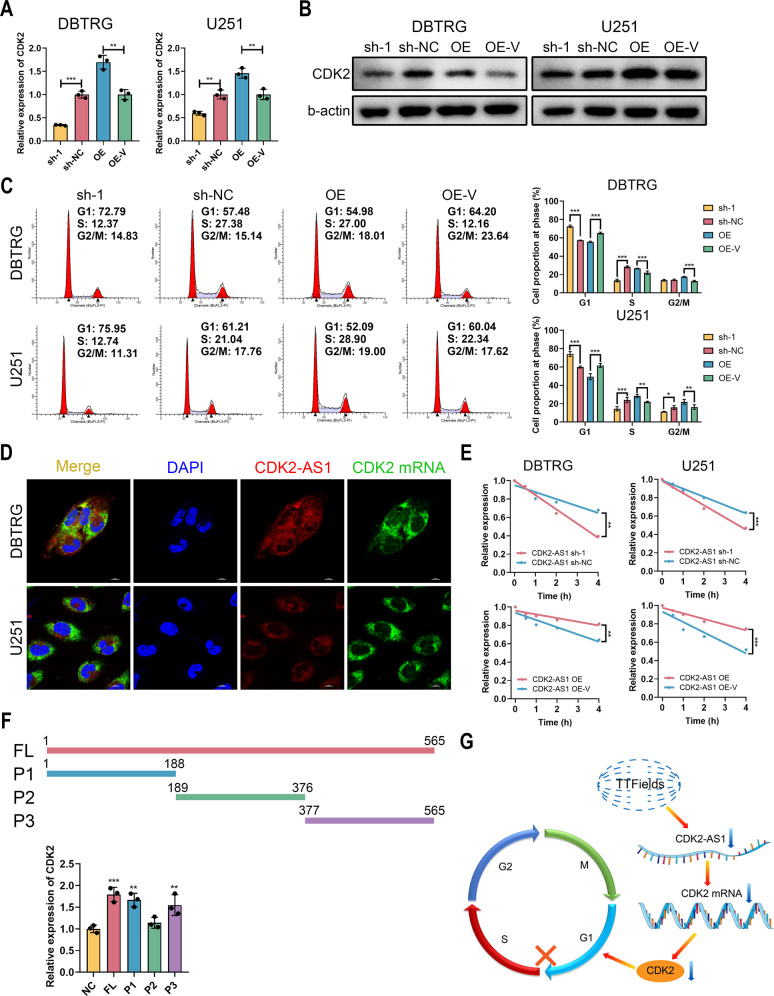
Knockdown of CDK2-AS1 induced cell cycle arrest via decreasing CDK2 mRNA stability. The mRNA (**A**) and protein **B** levels of CDK2 in DBTRG and U251 cells after the knockdown or overexpression of CDK2-AS1. **C** Cell cycle analysis of DBTRG and U251 cells after the knockdown or overexpression of CDK2-AS1. **D** FISH assay of CDK2-AS1 and CDK2 mRNA (scale bar = 10 μm). **E** The stability of CDK2 mRNA after the knockdown or overexpression of CDK2-AS1. **F** Presentation of truncated CDK2-AS1 and their effects on CDK2 mRNA expression. **G** Schematic presentation of CDK2-AS1 as the target of TTFields against GBM. **p < 0.01, ***p < 0.001.

As mentioned above, CDK2-AS1 was proposed as the potential target of TTFields against GBM, however, its underlying mechanism remained unclear. Firstly, we supposed that CDK2-AS1 might interact with some oncogenic proteins to regulate GBM cell proliferation. However, CHIRP assay failed to detect proteins that interacted with CDK2-AS1 (Supplementary Fig. S7). Previous studies indicated that as a major subtype of lncRNA, the antisense lncRNA was reported to regulate cancer development via enhancing or decreasing the mRNA stability of sense genes^12-15^. Since CDK2-AS1 was the antisense transcript of CDK2, a critical cell cycle-associated protein, we hypothesized that CDK2-AS1 might affect the stability of CDK2 mRNA. Results showed that the knockdown of CDK2-AS1 significantly decreased the mRNA and protein levels of CDK2 whereas its overexpression exerted opposite function (Fig. 8A, B, Supplementary Fig. S11). Moreover, after the knockdown of CDK2-AS1, the proportion of cells arrested at G1 phase significantly increased and those at S phase significantly decreased, whereas the promotion of CDK2-AS1 decreased G1-phase cells but increased S-phase cells (Fig. 8C). Further, FISH assay demonstrated the co-localization of CDK2-AS1 and CDK2 mRNA (Fig. 8D). The stability of CDK2 mRNA was significantly diminished after the knockdown of CDK2-AS1 but promoted by CDK2-AS1 overexpression (Fig. 8E). Specifically, when we truncate CDK2-AS1 into three segments (P1-P3), we found that P1 and P3 were responsible for promoting CDK2 mRNA expression (Fig. 8F). Therefore, we hypothesized that TTFields could reduce the expression of CDK2-AS1, which further decreased the expression of CDK2 via mitigating CDK2 mRNA stability and resulted in GBM cell cycle arrest (Fig. 8G).

## Discussion

In the past decade, only three therapies have been approved by the Food and Drug Administration (FDA) for GBM treatment, in which TMZ and bevacizumab are chemotherapy drugs that have multiple adverse effects on patients despite their efficacy in improving survival [13, 14]. TTFields is the novel therapy exhibiting promising efficacy in treating newly diagnosed and recurrent GBM [15]. However, molecular mechanism of TTFields remains to be further elucidated. Herein, our study demonstrated that the CL-301A could significantly suppress GBM cell proliferation and induce cell cycle arrest as well as cell apoptosis. By conducting transcriptional and proteomic sequencing, we found that TTFields mainly disrupted cell mitosis-related events such as DNA replication, spindle formation, microtube binding, nuclear division, etc., which was consistent with previous studies [5, 6]. Meanwhile, we revealed that TTFields could inhibit cell autophagy to promote cell apoptosis, and CDK2-AS1 was identified as the potential target of TTFields.

Since TMZ was established as the first-line therapy for GBM patients in 2005, no trial results or treatment have been broadly embraced with promising efficacy. Although immunotherapy has achieved great progress in the treatment of melanoma [16], urothelial carcinoma [17], non-small cell lung cancer [18, 19], and other cancers [20], its application in GBM remains to be a challenge. In a phase III randomized clinical trial (CheckMate 143), the programmed death-1 (PD-1) inhibitor, nivolumab, did not significantly prolong median OS of patients with recurrent GBM compared with bevacizumab (9.8 months vs 10.0 months) [21]. Therefore, the inspiring efficacy of TTFields brings new hope for GBM patients. Based on the antimitotic mechanism of TTFields, our research team developed the TTFields system (ASCLU-300 for human wear device and CL-301A for cell intervention device), and its efficacy was proven both in vitro and in vivo [10, 11]. Up to now, a prospective pilot study that evaluates the safety of ASCLU-300 in recurrent GBM has accomplished (ChiCTR2000032655, NCT04417933). Results indicate that ASCLU-300 is tolerable for GBM patients and its main adverse event is dermatitis (unpublished date), which is consistent with animal experiments and previous studies [6, 7, 10, 22–24]. Further, a large-scale randomized clinical trial that evaluates the efficacy and safety of ASCLU-300 in newly diagnosed supratentorial GBM is ongoing (ChiCTR2100047049). As for CL-301A system that was used in this study, our results demonstrated its efficacy in suppressing GBM cell proliferation and induce cell apoptosis.

After the validation of anti-GBM activity of CL-301A system, we would like to explore its molecular mechanism. However, few patients would receive surgery after TTFields intervention, which led to limited source of TTFields-treated specimens. Therefore, we used GBM cells to preliminarily explore the underlying mechanism of TTFields. Previous studies indicated that there were two mechanisms of TTFields against tumor cell proliferation: one was interrupting microtubule polymerization and subsequently leading to mitotic arrest; another one was inducing dielectrophoresis [5, 6, 25]. Similar to previous results, our study revealed that TTFields mainly affected proteins in the nucleus, and these proteins were associated with cell division, DNA replication, cell cycle, etc. Besides, nuclear condensation and cytoskeletal disturbance were detected in cells after TTFields intervention. Therefore, our results indicated that CL-301A could efficiently exert anti-GBM activity in parallel to NovoTTF-100A System.

Although the efficacy of TTFields has been demonstrated by the large-scale randomized clinical trial, but its molecular mechanism remains largely unknown. Shteingauz et al. revealed that AMPK-dependent autophagy is promoted as a survival mechanism against TTFields in glioma cells [26], which was consistent with our findings that the inhibition of autophagy significantly enhanced the anti-tumor activity of TTFields. Meanwhile, we noticed that the level of LC3II/I was promoted after TTFields and further increased after CHQ treatment in their results. In contrast, our study revealed that LC3II/I was increased after TTFields but not markedly changed if cells were pre-treated with CHQ. The main difference between Shteingauz et al. and our study was the timing of CHQ application. They added CHQ 3–4 h before TTFields treatment end, but our study added CHQ before the intervention. The different timing might lead to the different results. Regarding the difference, we hypothesized that GBM cells promoted autophagy in response to TTFields, which stimulated the transition of LC3I to LC3II. However, lysosomes-mediated degradation might be interrupted by TTFields. Since lysosome was responsible for autophagosome degradation [27, 28], its disorder would lead to the accumulation of LC3-II. And this result suggested decreased autophagic flux rather than induction of autophagy [12]. Meanwhile, the expression of p62, the protein that was largely degraded by autophagy [29, 30], was significantly promoted after TTFields intervention. Therefore, we proposed that TTFields could inhibit GBM cell autophagy. Nevertheless, we had the same result that the inhibition of autophagy could promote the efficacy of TTFields and autophagy was the survival mechanism of GBM cells in response to TTFields. However, whether autophagy inhibition would promote or mitigate the efficacy of TTFields remained controversial since previous studies indicated that the inhibition of autophagy would ameliorate TTFields-mediated cell death [31, 32]. Therefore, additional experiments were needed to explore and characterize the mechanism of autophagy during TTFields intervention.

Among identified differentially expressed RNAs and non-coding RNAs, we would like to find a potential target that mediated the anti-tumor activity of TTFields. Emerging evidence suggested that lncRNAs played critical roles in regulating biological process and in cancer development [33], and its role in TTFields remained unknown. Therefore, based on the anti-mitotic activity, we select cell cycle-related genes to construct a mRNA-lncRNA co-expression network, in which CDK2-AS1 was selected after preliminary experimental results. CDK2-AS1 belongs to a a type of lncRNAs termed antisense that accounts for a substantial proportion. Previous studies indicated that the antisense lncRNA was reported to promote cancer development via enhancing or decreasing the mRNA stability of sense genes [34–37]. For example, LDLRAD4-AS1 could decrease the mRNA stability of LDLRAD4 to promote colorectal cancer metastasis [34]. Moreover, FOXC2-AS1 could increase the mRNA stability of FOXC2 to promote doxorubicin resistance in osteosarcoma [37]. Similarly, HOXD-AS1 could recruit PRC2 to decrease the transcription of HOXD3, thus decreased the proliferation and migration of colorectal cancer [36]. Herein, our study revealed that CDK2-AS1 might be the target of TTFields since its knockdown could enhance the anti-tumor activity of TTFields against GBM cells. Mechanistically, CDK2-AS1 could enhance the mRNA stability of CDK2. Therefore, we hypothesized that TTFields could decrease the expression of CDK2-AS1, which could reduce the mRNA stability and the expression of CDK2, leading to cell cycle arrest.

Although we have demonstrated that inhibition of autophagy can enhance the anti-tumor effect of TTFields, the main target and molecular mechanism of TTFields in regulating autophagy remains unknown. Besides, since our study was conducted using immortal cells, the alternation of tumor microenvironment after TTFields could not be characterized. Our previous work established an integrated system using patient-derived glioma cerebral organoids and xenografts that could simulate glioma microenvironment [38]. The exploration of effect of TTFields on this integrated system would provide better understanding of TTFields in treating GBM.

To sum up, through whole-transcriptional and whole-proteomic sequencing, our study drew the expression landscape of DEPs and RNAs including coding and non-coding RNAs. The combination of bioinformatic analyses and experiments revealed that inhibition of autophagy could enhance the anti-tumor effects of TTFields. Besides, TTFields might target at CDK2-AS1 to induce cell cycle arrest via diminishing CDK2 mRNA stability. Our study would provide novel insights into the therapeutic mechanisms of TTFields against GBM.

## Materials and methods

### Cell culture and TTFields intervention

DBTRG-05MG (DBTRG) and U251 cells were used in this study. DBTRG cells were cultured in RMPI-1640 with 10% fetal bovine serum (FBS, ExCell Bio, China) and U251 cells were maintained in DMEM with 10% FBS. The specific TTFields device for cell culture was provided by Antai Kangcheng Biotechnology Co., Ltd. Cells were treated with TTFields intervention as previously described [11]. In brief, 5 × 10^4^ cells were seeded in 20 mm coverslip and then placed in the TTFields matched boxes. The parameter was set with the frequency of 200 kHz and the intensity of 1.5 V/cm. The flow chart of TTFields intervention was shown in Supplementary Fig. S1.

### Reagents

Cells were treated with DMSO (Sigma, USA), AC-DEVD-CHO (5 mM, MCE, HY-P1001), Z-VAD-FMK (Z-VAD, 20 mM, MCE, HY-16658B), Autophinib (1 μM, MCE, HY-101920), 3-Methyladenine (3-MA, 5 mM, MCE, HY-19312), Rapamycin (Rapa, 100 nM, MCE, HY-10219), Chloroquine (CHQ, 20 μM, MCE, HY-17589A) with indicated concentration before TTFields intervention.

### Cell viability assay

The Cell Counting Kit CCK-8 (Dojindo, Japan) was used to evaluate cell viability. Cells with intervention or not were seeded in 96-well plates at 2000 cells per well. Then 10 µl CCK-8 reagent was added and the absorbance at 450 nm was measured. For cells after TTFields intervention, the coverslip was placed in 12-well plate and 100 µl CCK-8 reagent to measure its viability.

### 5-Ethynyl-2’-Deoxyuridine (EdU) assay

Cells were incubated with 50 μM EdU solution for 3 h and then fixed using 4% paraformaldehyde. The Edu was stained with Apollo 567 and Hoechst 33342 was applied to stain the cell nuclei. Cells were detected using Eclipse Ti2-A fluorescence microscope (Nikon, Japan).

### Flow cytometry

Cell apoptosis and cell cycle were analyzed by flow cytometry. Cell apoptosis was evaluated using FITC Annexin V Apoptosis Detection Kit I (BD Biosciences, 556547). The APC-conjugated Annexin V was used for cells transduced with GFP lentivirus. For cell cycle analysis, cells were fixed using 70% cold ethanol at −20 °C overnight. Then the Cell Cycle and Apoptosis Kit (UE, C6031S) was used for staining and cell cycle was estimated on BD FACSCanto II.

### Immunofluorescence

Cells were fixed with 4% paraformaldehyde and stained with PI. Cells were detected using Eclipse Ti2-A fluorescence microscope (Nikon, Japan). For phalloidin staining, cells were incubated with 0.5% Triton X-100 PBS solution for 10 min. Then they were detected using ZEISS LSM880 confocal microscopy.

### Tandem mass tag (TMT)-labeled proteomics and LC-MS/MS analysis

The TMT-labeled proteomics was provided by Shanghai Applied Protein Technology (Shanghai, China). Briefly, the protein peptides were labeled sing TMT reagent and fractionated by strong cation exchange (SCX) chromatography. Then they were subjected to LC-MS/MS analysis on a Q Exactive mass spectrometer. Then the MS raw data of each sample were identified and quantified using Proteome Discoverer 1.4. The subcellular localization of proteins was predicted using CELLO (http://cello.life.nctu.edu.tw/). The protein domain was annotated using InterProScan software from the Pfam database (https://pfam.xfam.org). Differentially expressed proteins (DEPs) were screened using “limma” R package with the cut-off of |fold change|>1.2 or <0.8 and *p* value < 0.05.

### RNA sequencing and bioinformatics analyses

RNA sequencing including mRNAs, miRNAs, lncRNAs and circRNAs was performed using the Shbio Human (4 × 180 K) ceRNA array provided by Shanghai Biotechnology Coporation, China. To construct competing endogenous RNA (ceRNA) network, the potential interaction between circRNA-miRNA or lncRNA-miRNA and miRNA-mRNA was downloaded from ENCORI online webtool (http://starbase.sysu.edu.cn/). Pearson correlation analysis was used to screen correlated circRNA/lncRNA-mRNA. After intersected with differentially expressed mRNAs, miRNAs, lncRNAs and circRNAs, the ceRNA network was constructed using Cytoscape 3.8.0. The gene set of cell cycle was downloaded from Kyoto Encyclopedia of Genes and Genomes (KEGG, https://www.genome.jp/kegg/). The lncRNA-mRNA co-expression network was constructed based on correlation efficient and *p* value using Cytoscape 3.8.0. The protein-protein interaction (PPI) was analyzed on STRING website (http://string-db.org/) and visualized using Cytoscape 3.8.0. The “MCODE” was used to identify key modules within the PPI network. Differentially expressed mRNAs (DEmRNAs), miRNAs (DEmiRNAs), lncRNAs (DElncRNAs), and circRNAs (DEcircRNAs) were identified using “limma” R package with the cut-off of |fold change| > 2.0 or <0.5 and *p* value < 0.05.

### Enrichment analysis

Enrichment analysis was conducted using “clusterprofiler” R package including Gene Ontology (GO) and KEGG, in which GO contained three items: biological process (BP), cellular component (CC) and molecular function (MF). Those with false discovery rate (FDR) ≤ 0.05 were selected.

### Quantitative real-time PCR (qRT-PCR)

Total RNA was extracted by TRIzol Reagent (Invitrogen, USA). RNA of nucleus and cytoplasm was extracted using PARIS Kit (Invitrogen, USA). Then the cDNA was synthesized using PrimeScript RT reagent Kit (Takara, RR047A). The qRT-PCR was conducted on QuantStudio 5 Real-Time PCR System (ABI, Thermo) as follows: 95 °C for 5 min; 40 cycles of 95 °C for 10 s and 60 °C for 30 s. The mRNA expression was normalized to GAPDH and calculated by the 2^−ΔΔCt^ method. The primers used in this study was listed in Supplementary Table S6.

### Western blot

The total protein was extracted using RIPA lysis buffer (Beyotime, China) with Protease inhibitor Cocktail (MCE, China). The protein was separated in 10% or 12.5% SDS-PAGE gel and transferred to the PVDF membrane (Millipore, USA). After the blockade with 5% skim milk for 1 h at room temperature, the membrane was incubated with primary antibodies at 4 °C overnight. The secondary antibody HRP-conjugated anti-rabbit or anti-mouse IgG was applied for 1 h at room temperature and the protein content was detected using an enhanced chemiluminescence system. The primary antibodies used in this study was listed in Supplementary Table S6.

### Plasmid construction and cell transfection

The full-length cDNA sequence of human lnc-CDK2-AS was synthesized and cloned into plasmids provided by GeneChem (Shanghai, China). Three shRNAs were designed targeting lnc-CDK2-AS and cloned into plasmids provided by GeneChem (Shanghai, China). The sequence of three shRNAs were listed in Supplementary Table S6. The lentivirus was produced in HEK-293T cells that were transfected with the lnc-CDK2-AS overexpression vector and package vectors using Lipofectamine 3000 (Invitrogen, Thermo).

### Estimation of mRNA stability

The Actinomycin D was used to block the synthesis of RNA. DBTRG or U251 cells transduced with sh-CDK2-AS1-1 or overexpressed lentivirus were seeded in the six-well plate. When cells grew to 50% confluence, they were treated with 10 μg/ml Actinomycin D for 0, 0.5, 1, 2, and 4 h, respectively. Then the total RNA was extracted and subjected to qRT-PCR. The mRNA stability was estimated according to previously published paper [39].

### Fluorescence in situ hybridization (FISH)

The probes for CDK2-AS1 and CDK2 mRNA was synthesized by RiboBio (Guangzhou, China). The details of probe sequence were listed in Supplementary Table S6. DBTRG and U251 cells were fixed with 4% paraformaldehyde and treated with PBS containing 0.5% Triton X-100. Then cells were incubated with probes at 37 °C overnight. After three times of washing using saline sodium citrate (SSC), cells were stained with DAPI. Then the fluorescence was detected using Nikon Eclipse C2 confocal microscope.

### Statistical analysis

Data analyses and visualization were conducted using GraphPad Prism 8 and R 3.6.3. The difference comparison was performed using Student’s *t* test or one-way ANOVA between two or more than two groups. The *p* value < 0.05 was considered statistically significant.

## Supplementary information

Supplementary figures

Table S1

Table S2

Table S3

Table S4

Table S5

Table S6

Nature reproductability form

## Data Availability

All data generated or analyzed during this study are included in this published article and its supplementary information files.
