# Correction: Whole transcriptome and proteome analyses identify potential targets and mechanisms underlying tumor treating fields against glioblastoma

**DOI:** 10.1038/s41419-022-05228-3

**Published:** 2022-09-19

**Authors:** Shengchao Xu, Chengke Luo, Dikang Chen, Lu Tang, Ling Chen, Zhixiong Liu

**Affiliations:** 1 Department of Neurosurgery, Xiangya Hospital, Central South University, Changsha, 410008 Hunan China; 2 National Clinical Research Center for Geriatric Disorders, Xiangya Hospital, Central South University, Changsha, China; 3 Hunan An Tai Kang Cheng Biotechnology Co., Ltd, Changsha, China; 4 Department of Anesthesiology, Xiangya Hospital, Central South University, Changsha, 410008 Hunan China; 5 Department of Neurosurgery, Chinese People’s Liberation Army of China (PLA) General Hospital, Medical School of Chinese PLA, Institute of Neurosurgery of Chinese PLA, 100853 Beijing, China

**Keywords:** CNS cancer, Cancer therapy

Correction to: *Cell Death and Disease* 10.1038/s41419-022-05127-7, published online 18 August 2022

The original version of this article contained an error in the affiliations. The authors forgot to add the affiliation of “National Clinical Research Center for Geriatric Disorders, Xiangya Hospital, Central South University, Changsha, China” for Shengchao Xu, Chengke Luo, and Zhixiong Liu. The authors apologize for the error. The original article has been corrected.

