# Supplementary material for: Whole transcriptome and proteome analyses identify potential targets and mechanisms underlying tumor treating fields against glioblastoma
Source: Cell Death Dis. 2022 Aug 18;13(8):721. doi: 10.1038/s41419-022-05127-7 (PMC9388668; doi:10.1038/s41419-022-05127-7)
Supplement: Supplementary file 1 — Supplementary figures [file 41419_2022_5127_MOESM1_ESM.pdf]

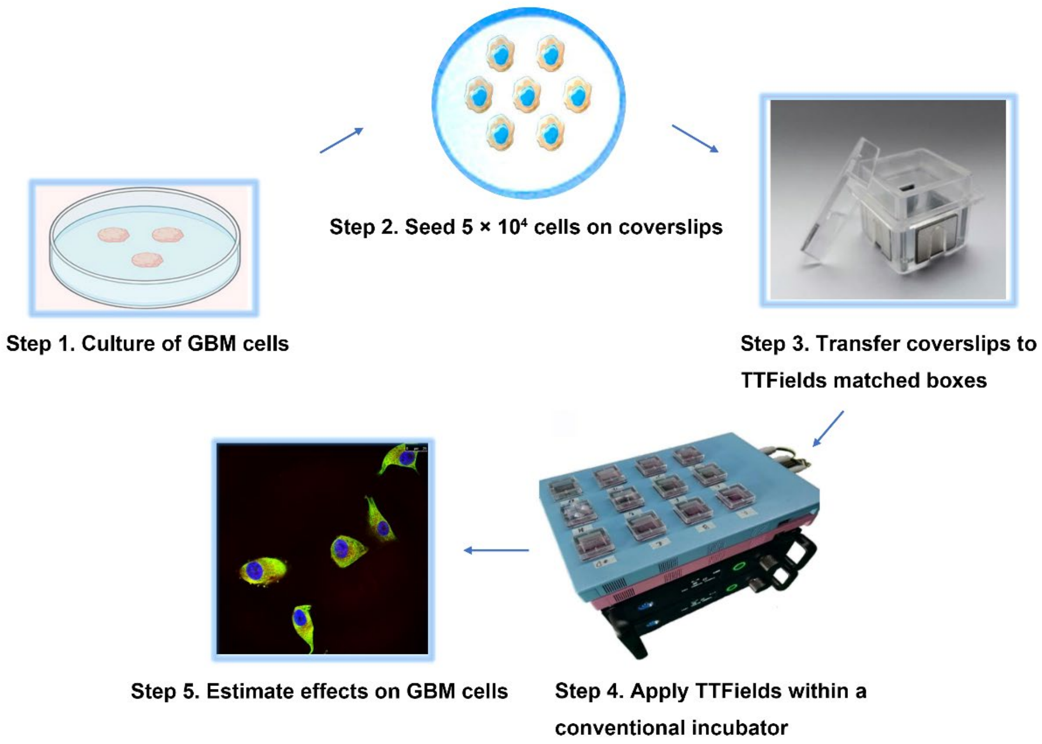

Figure S1. Flow chart of TTFields intervention.

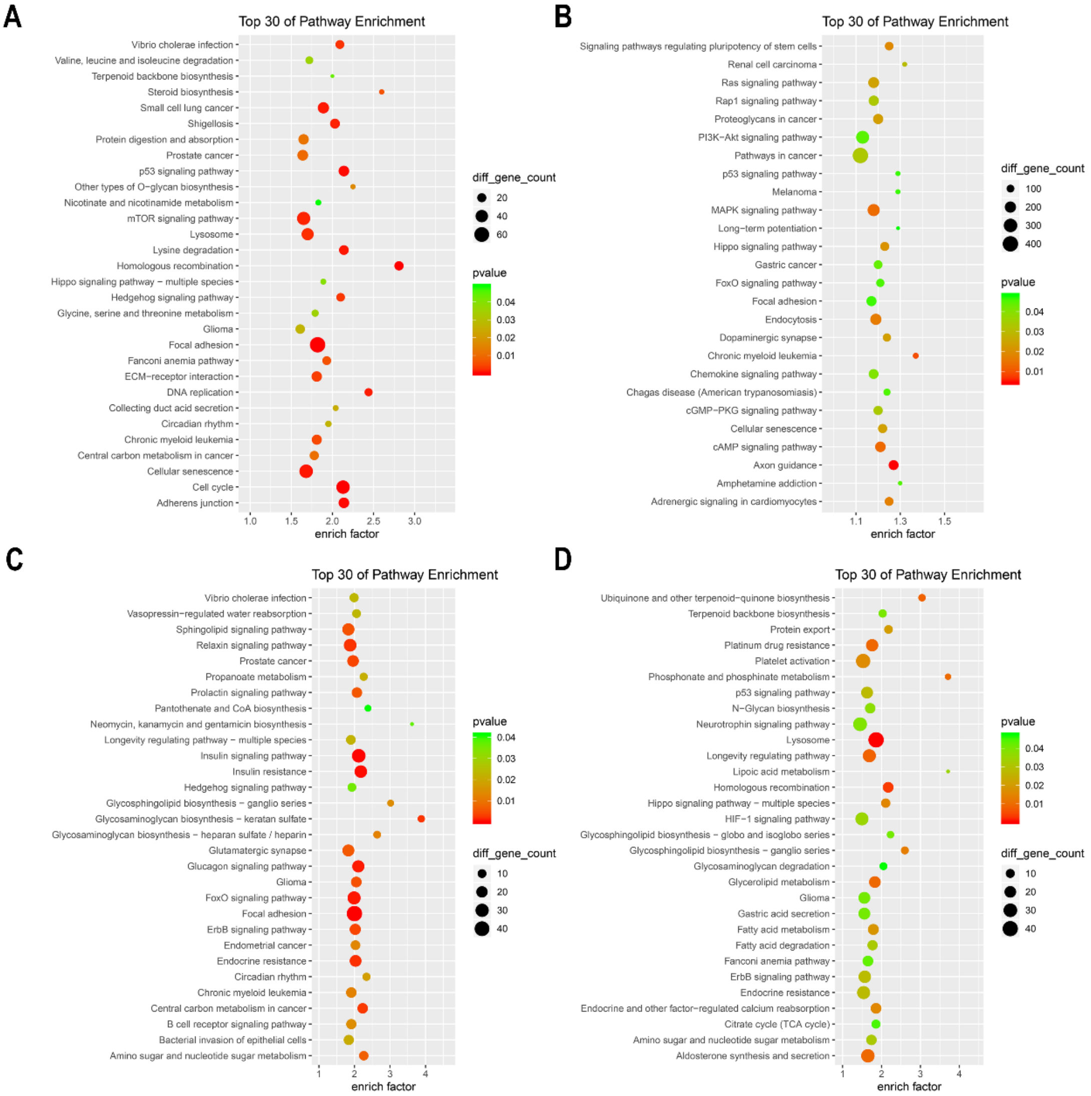

Figure S2. Functional enrichment of parent genes of DEcircRNAs and target genes of DEmiRNAs and DElncRNAs. A-D. KEGG enrichment analysis of parent genes of DEcircRNAs (A), target gene of DEmiRNAs (B), DElncRNAs cis-regulated genes (C), and DElncRNAs trans-regulated genes (D).

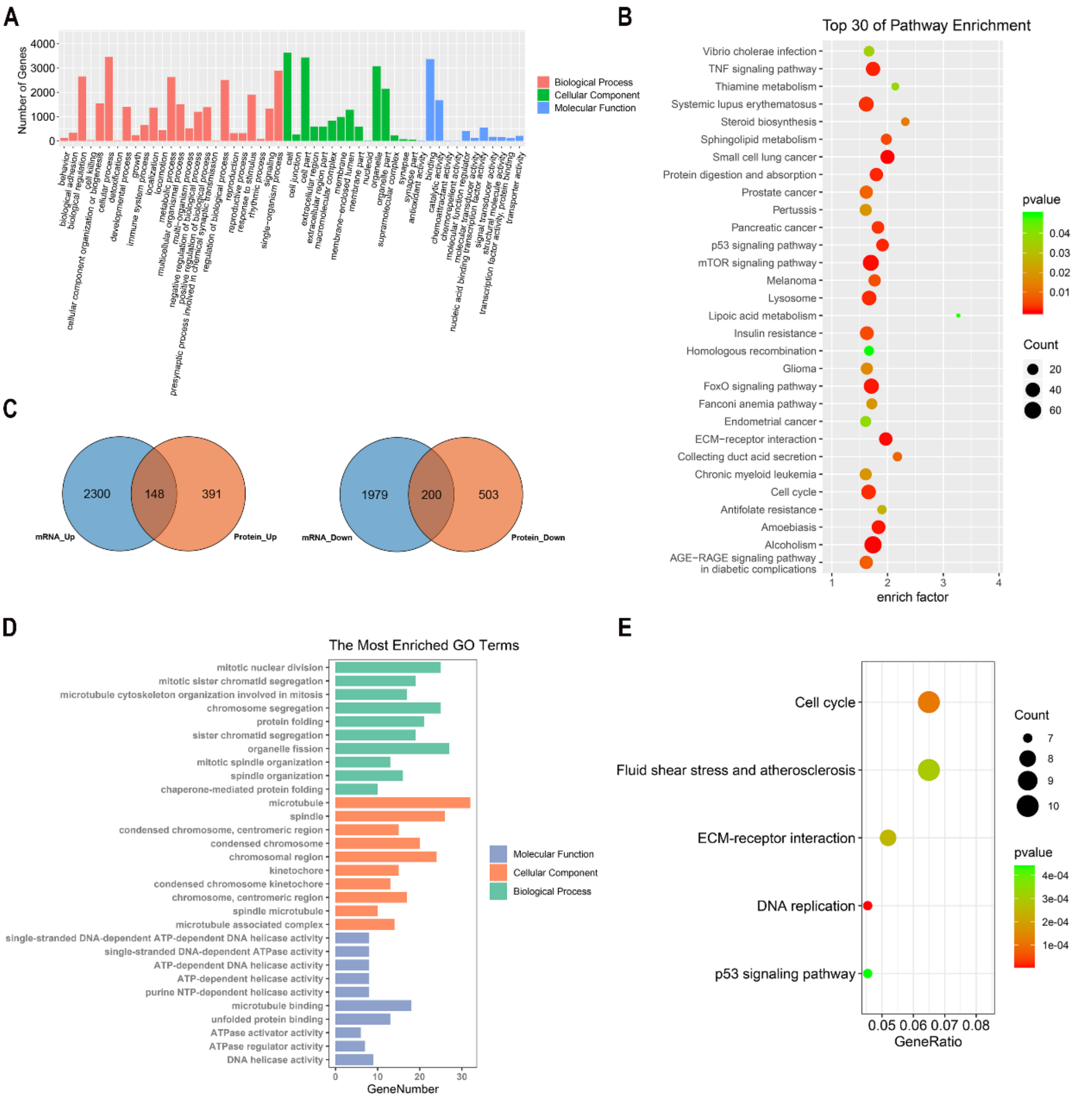

Figure S3. Functional enrichment of co-expressed DEPs-DEmRNAs. A-B. GO enrichment analysis of DEmRNAs. **C.** The overlapped genes between upregulated or downregulated DEmRNAs and DEPs. **D-E.** GO (D) and KEGG (E) enrichment analyses of overlapped genes.

Protein	mRNA	Number
↓	↓	200
↑	↑	148
—	↓	150
↓	—	174
↑	—	170

Figure S4. The number of DEPs-DEmRNAs with different expression patterns.

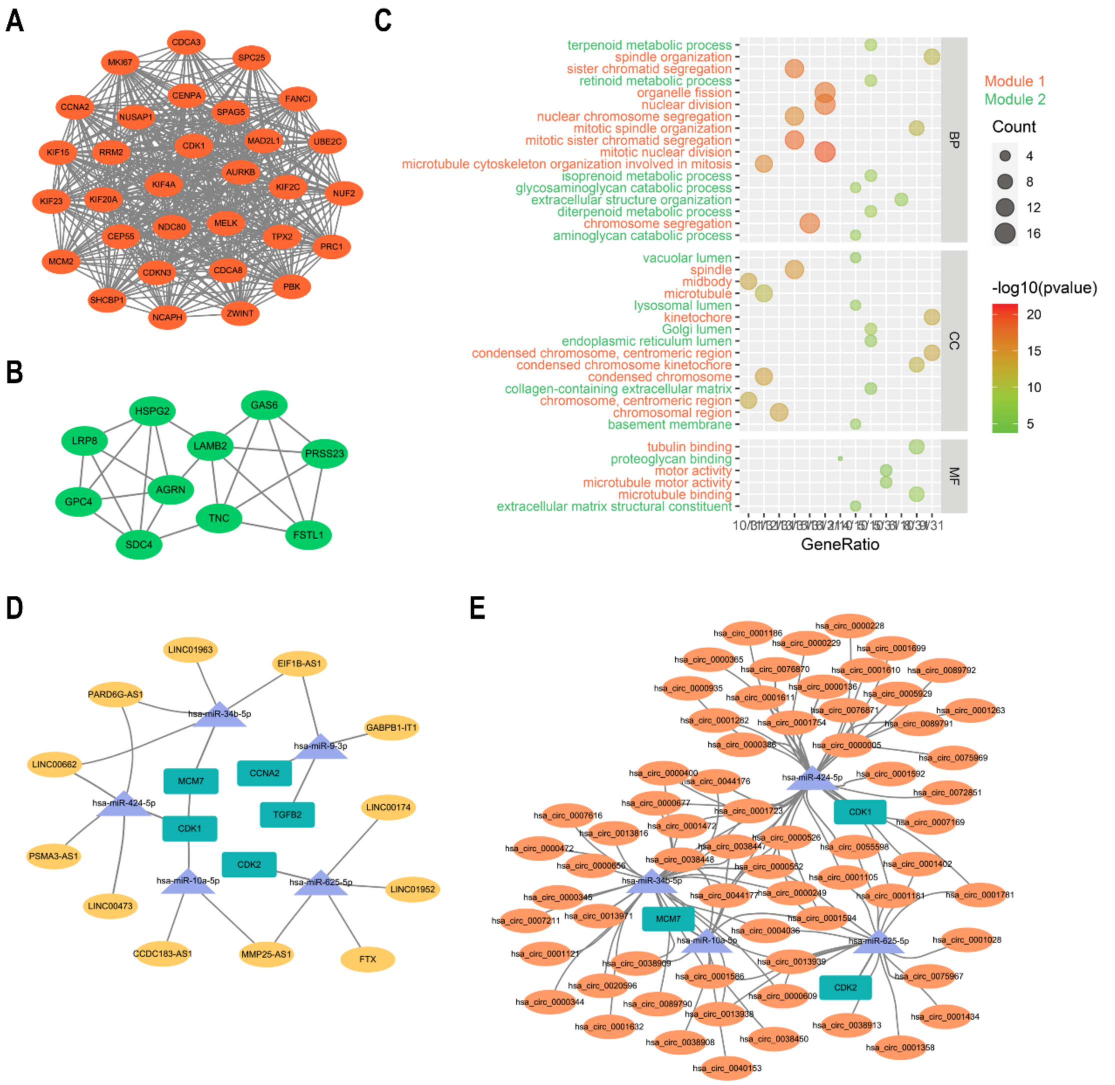

Figure S5. Identification of key modules of downregulated proteins and construction of ceRNA network. A-C. Two modules of downregulated proteins (A-B) and GO enrichment analysis (C). **D.** Construction of lncRNA-miRNA-mRNA network. **E.** Construction of circRNA-miRNA-mRNA network.

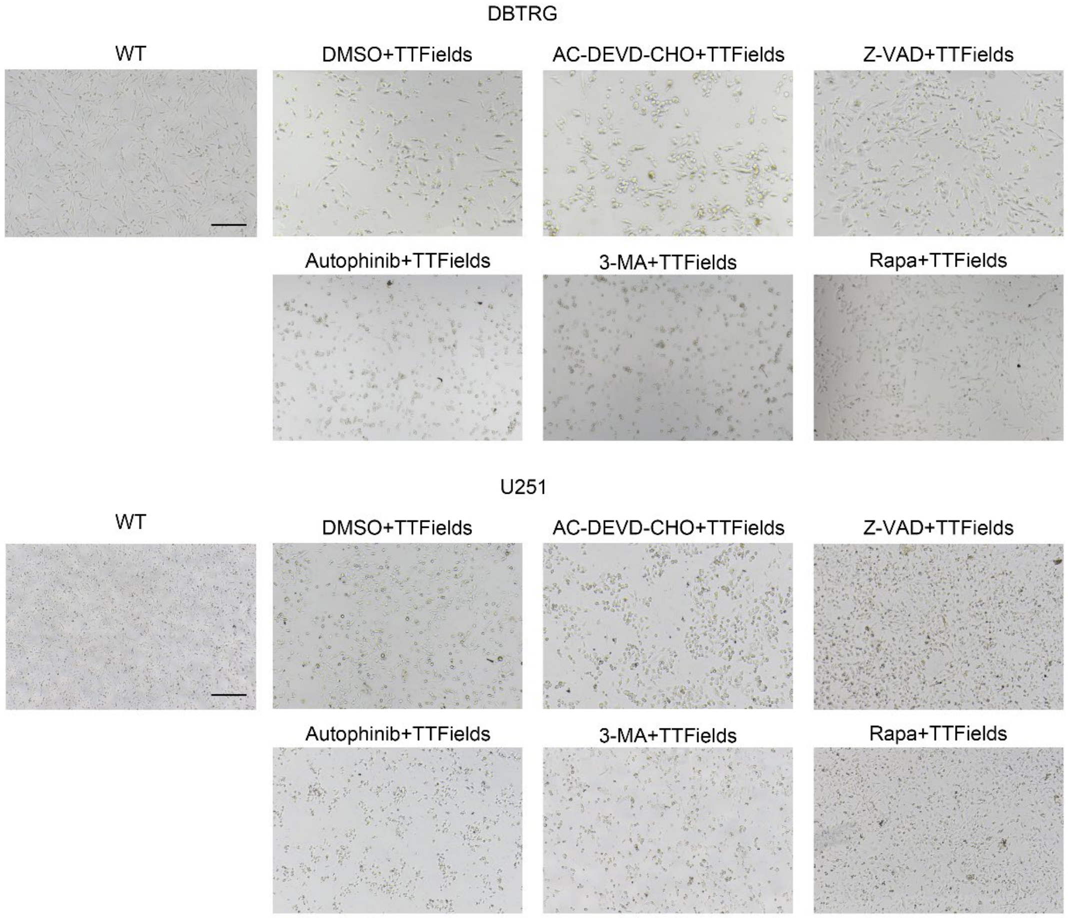

Figure S6. Cell morphology of DBTRG and U251 cells. DBTRG and U251 cells that were pretreated with DMSO, 5 mM AC-DEVD-CHO, 20 mM Z-VAD, 1 μM Autophinib, 5 mM 3-MA, or 100 nM Rapa for 12 h and subsequent TTFields intervention for 48 h.

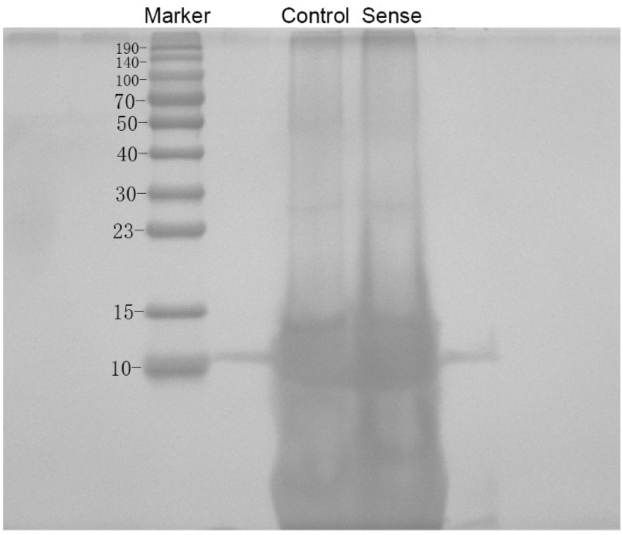

Figure S7. Silver-staining of proteins extracted using CDK2-AS1 sense or control probes by CHIRP assay.

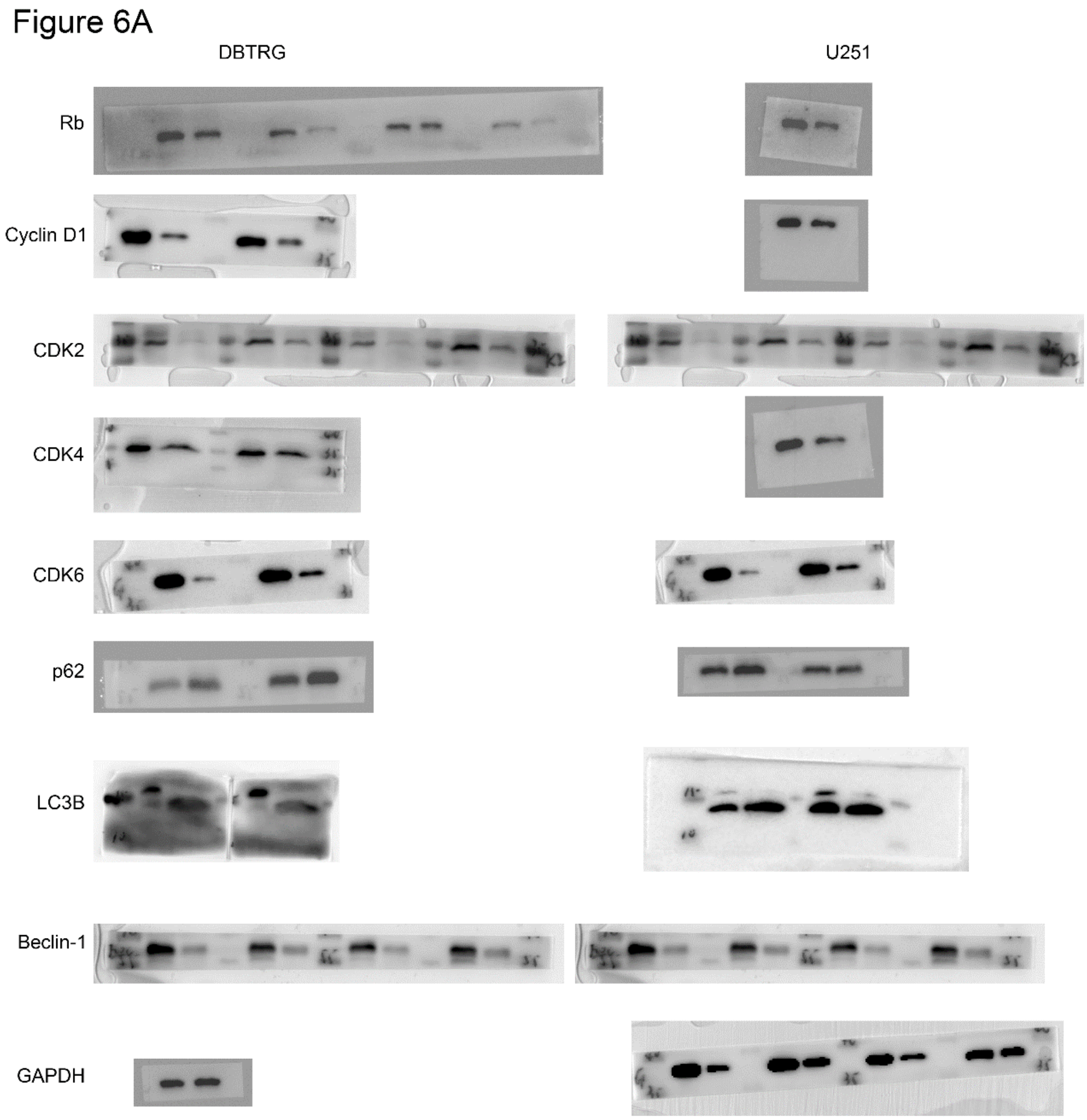

Figure S8. Original blot of Figure 6A.

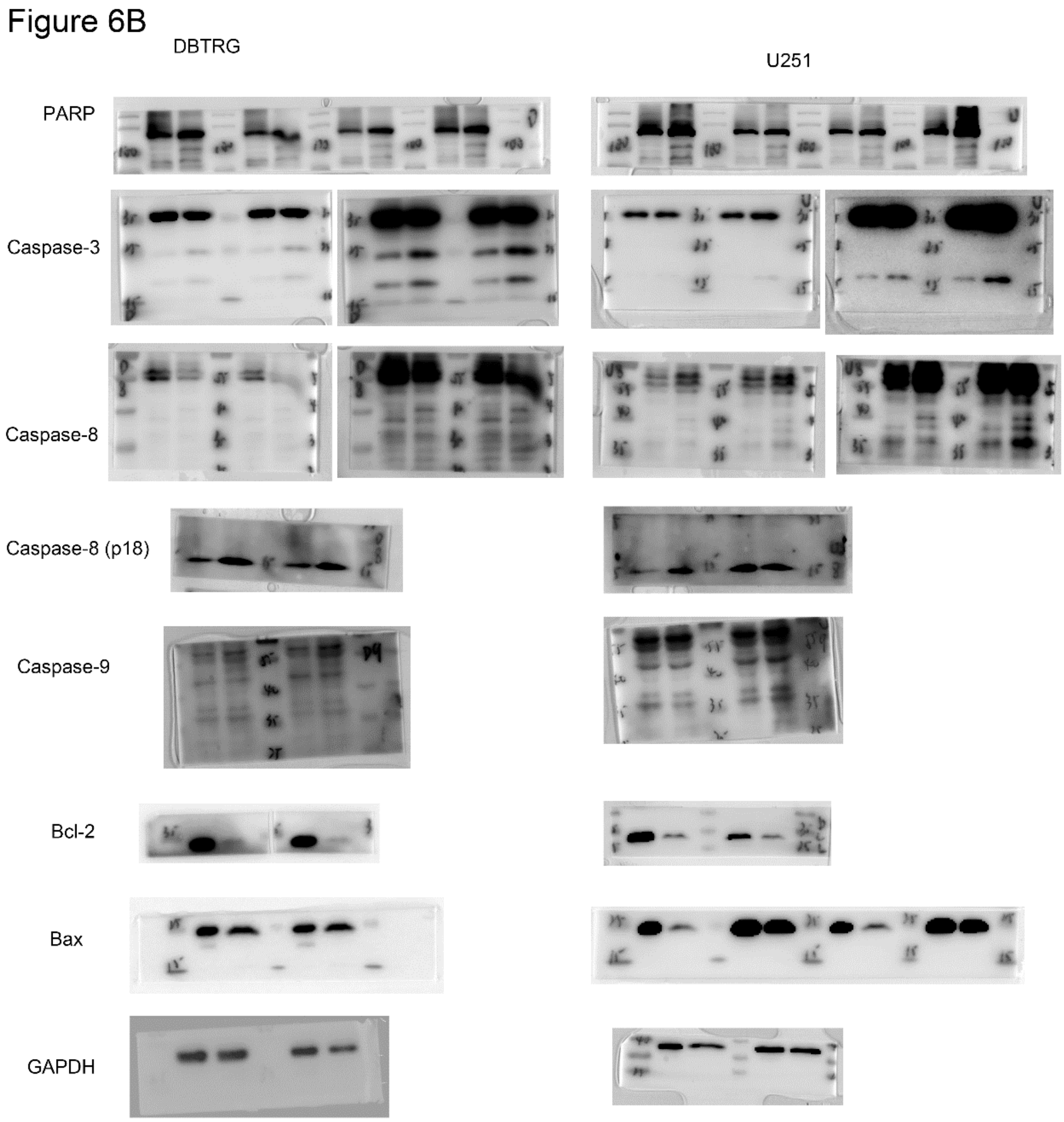

Figure S9. Original blot of Figure 6B.

Figure 6C

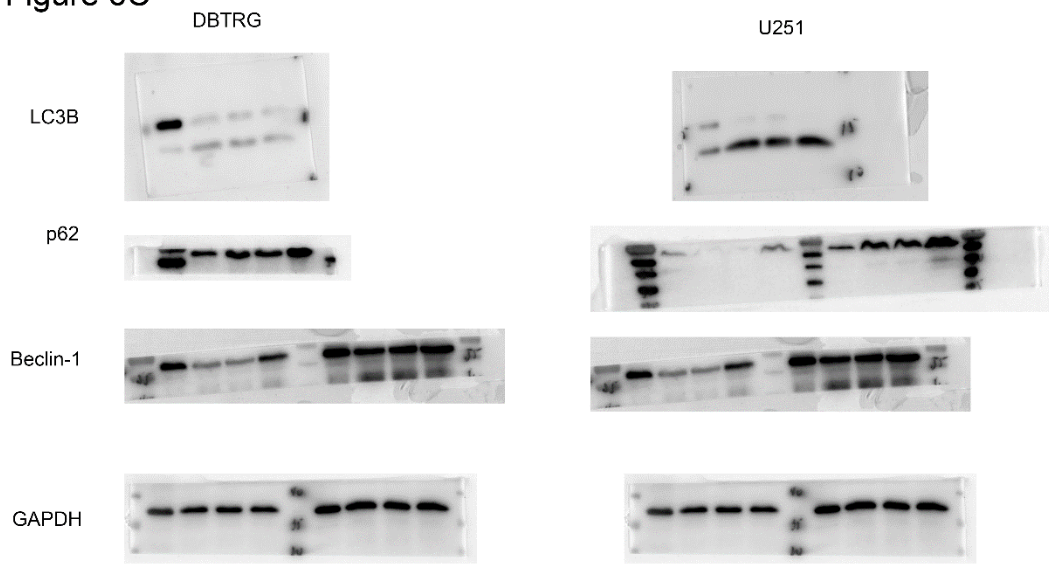

Figure S10. Original blot of Figure 6C.

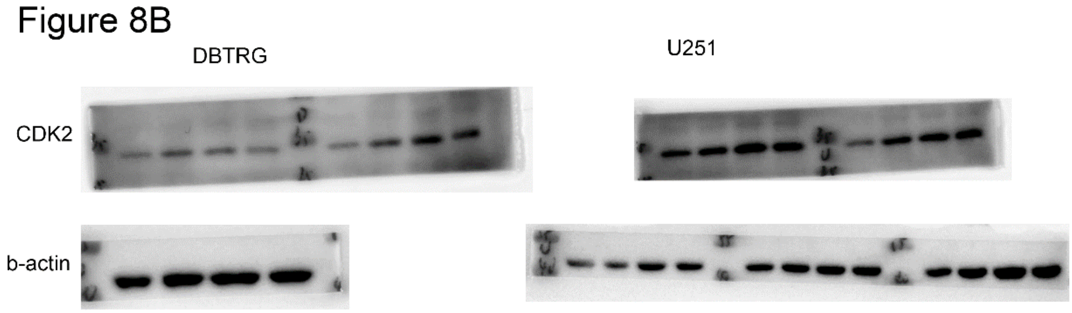

Figure S11. Original blot of Figure 8B.
